# Modern Sociology

**Published:** 1900-02-17

**Authors:** 


### Modern Sociology.

### TRUCK ACTS AT HOME AND ABROAD.

The Truck Acts are those designed to prevent the payment of wages in any other form than coin of the realm. At the present day there are few places where the payment of wages in money would be anything but an inconvenience to both employers and employed; but in the beginning of the industrial era this was not the case. Especially before steam became general, when machinery was used, but was worked by water power, factories were often erected in lonely and inaccessible spots. In time, no doubt, trade would follow manufacture, and set up shops for the retailing of the necessaries of life; but communication was not good, railways were not, and thus a man might be earning good wages and yet have less of the comforts of life than one who was making a much smaller sum in a town. Under these circumstances, it was a convenience when the master set up a store where the workers might purchase what they needed. Unfortunately, the idea soon dawned upon the founder of the store that here was another source of income. He might charge more than the ordinary retail price for the goods he supplied; he might charge the price of the best quality of articles for those of inferior quality; he might make it, formally or informally, a *sine quâ non* that all his workers should trade at the factory store. All these things happened, and the result was that the worker was practically cheated of great part of his wages, forced to give a famine price at his master's store for things which he could have bought cheaper elsewhere; and this state of things continued even after ordinary shops had been set up in the neighbourhood of the factory. It was for this reason that the first Truck Act was passed in 1831.

The Truck Acts declare that workmen must be paid their wages in full in the current coin of the realm, but bank-notes or cheques payable on demand may be accepted in place of actual coin. Even should the workman accept part of his wages in kind he may sue for the payment of his full wages in money, and the employer cannot bring forward as a set-off any goods supplied by himself or any store in which he is interested. To the advantage which this clause gives the workman is added another, for an employer cannot sue the workman for the value of any goods which have been furnished to him as part of his wages. As, however, an employer may, for the sake of friendship or patronage to the storekeeper, wish his employés to trade at a shop in which he has no pecuniary interest, it is further enacted that all contracts between employer and employed imposing conditions regarding the place where, and the manner in which wages are to be expended, are illegal and void. This injunction formed part of the original Truck Act of 1831; and in 1887 a further limitation was added, viz., that no employer may, himself or through his agent, dismiss a workman because of the place or way in which he has expended his wages. In Germany the provisions of the Truck Act are substantially the same, but greater allowance is made for the chance of a factory being in a place where shops are rare, and this allowance is all to the advantage of the worker, for his employer is permitted to provide him with food, but only at cost price. A similar regulation appears in the Austrian code. In the latter it is very specifically stated that employers may not supply workers on credit with goods, with certain specified exceptions, and are particularly forbidden to supply them with intoxicating drinks. In Continental countries, as well as in our own, it is forbidden to pay wages in a public-house. In 1872 this restriction was introduced in the coal-mining industries, and in 1883 it was extended to all other industries.

There are, however, certain remissions of this refusal to let the employer interfere with the expenditure of the workman's wages. If a special contract is entered into, the employer may furnish, as a part of wages, medical supplies and attendance; while fuel, materials, and tools to be used in the trade followed, provender for beasts of burden, food prepared and eaten in the employer's house, and a dwelling-house, may also be reckoned in deciding wages. Agricultural labourers may have food, non-intoxicating drink, a cottage and garden, and various privileges reckoned as part of their remuneration. In agriculture more than in any other industry primitive conditions prevail, and it is clear that in many cases it will be for the advantage of both parties to make and receive payments in kind.

The provision of a house is, however, occasionally a source of trouble in other industries. It is often necessary in establishing an industry in a new place—for example, in sinking a coal pit on a lonely moor—for the employer to erect houses for his workers. No speculative builder will take the risk of building houses which may, if the business does not succeed, soon be untenanted. But there is no doubt that this provision of houses gives the employer a power over his men. If they strike for any reason, he is obviously within his rights in evicting them. The houses are intended for his workers, not for mere tenants. At the same time, it is an undoubted hardship for a man to be turned out of his home, often on very short notice, because of a dispute with his master. Where a choice exists, one often finds workmen prefer to pay a larger rent for a house inferior to those which his employer provides, in order to preserve his complete independence. Much more rarely is there any trouble over medical attendance being contracted for, and paid by deductions from wages. The workers elect their doctor by their own vote, though, of course, the minority have to fall in with the choice of the majority, and pay for the one whom the latter choose. On the whole there is not much grumbling about the arrangement, while on the other hand it makes it possible for a doctor to be got for a place where no man would be willing to set up and take his chance, with the knowledge that if he began to make a satisfactory practice a competitor would come in to share what was enough for one but not enough for two.

The provisions of the Truck Acts do not and cannot apply to domestic servants, male or female. Their food and lodging form an essential part of their wages. It is this fact that makes inquiries into the wages of servants so futile. A large wage is only one factor in a good situation.

